# Antibiotic Prescribing in Patients Presenting with COVID-19: A Closed-Loop Audit with a Targeted Educational Intervention in a South Australian Hospital

**DOI:** 10.3390/jcm15010239

**Published:** 2025-12-28

**Authors:** Robert Yarham, Colette Dignam, Dylan Toh

**Affiliations:** 1 Department of Health and Medical Sciences, University of Adelaide, Corner of George Street and North Terrace, Adelaide, SA 5000, Australia; colette.dignam@sa.gov.au; 2 General Medicine Department, Royal Adelaide Hospital, Port Road, Adelaide, SA 5000, Australia; dylan.toh@sa.gov.au

**Keywords:** COVID-19, coronavirus, bacterial coinfection, pneumonia, antimicrobial stewardship, quality improvement, education intervention, antibiotic prescribing

## Abstract

**Background/Objectives**: Antimicrobial resistance is an escalating public health threat. Despite the low prevalence of community-acquired bacterial coinfection (CABC) in patients presenting with COVID-19, antibiotics are frequently prescribed. We evaluated antibiotic prescribing for suspected CABC in adults admitted with COVID-19 to a metropolitan referral hospital in South Australia and the impact of a targeted, locally developed educational intervention. **Methods**: We conducted a closed-loop retrospective audit of consecutive adult COVID-19 non-intensive care unit admissions to the General Medicine Unit over a six-month period (5 February–6 August 2024), totalling 126 episodes after exclusions. Antibiotic prescribing rates were compared before and after implementation of targeted education to admitting clinicians. This was delivered via guideline-focused presentations at unit meetings, emails and posters in key clinical areas detailing the consensus recommendation, and brief electronic reminder messages. Prescribing appropriateness was inferred from adherence to the consensus recommendation, which included predefined criteria relating to total leukocyte count, neutrophilia, and radiological findings. Statistical comparisons were performed using appropriate statistical tests for categorical and continuous variables. **Results**: Following the intervention, antibiotic prescribing decreased from 26% to 22% of admissions (*p* = 0.57), and prescribing among patients who did not meet the specified criteria fell from 16% to 6% (*p* = 0.30). The proportion of prescriptions meeting the consensus criteria increased from 62% to 80% (*p* = 0.43). **Conclusions**: Baseline antibiotic use was substantially lower than commonly published rates but still exceeded expected CABC prevalence. Targeted education and simple, locally adaptable guidance may support improved antimicrobial stewardship in COVID-19 admissions.

## 1. Introduction

Antimicrobial stewardship is a critical component in preventing the emergence and spread of antimicrobial resistance (AMR) and multidrug-resistant pathogens [1,2]. Antimicrobial resistance is associated with increased morbidity and mortality—over 700,000 deaths globally per year [1]—as well as prolonged hospitalisation, more frequent and severe treatment complications, and substantially higher healthcare resource utilisation [1,2,3]. Overuse of antibiotics is a key driver of AMR, increasing selection pressure on bacteria and promoting the dominance of resistant strains [3,4].

Coronavirus disease 2019 (COVID-19) represents an important setting of antibiotic overuse. Numerous studies have shown that the rate of community-acquired bacterial coinfection (CABC) in adult patients admitted to non-intensive care wards with COVID-19 is approximately 3–12% [5,6,7,8,9,10,11]—far lower than antibiotic prescribing rates, which often exceed 50% and approached 100% in some regions during the early stages of the pandemic [2,5,6,9,12]. Higher reported rates of coinfection in some large studies likely reflect broader case definitions (such as inclusion of secondary or non-bacterial infections, or absence of a clear definition) [12,13,14] and differing populations, including cohorts with a very high proportion of intensive care unit (ICU) or paediatric patients [2,7].

Limited data exist regarding the rate or appropriateness of antibiotic prescribing for CABC—defined as pulmonary bacterial coinfection present at the time of admission in those with confirmed COVID-19—within our institution. This audit aimed to review antibiotic prescribing practices in this setting and to provide targeted education for admitting clinicians, with the goal of reducing unnecessary antibiotic use in patients presenting with COVID-19.

## 2. Materials and Methods

This was a Quality Improvement closed-loop retrospective clinical audit conducted at a major metropolitan referral hospital in South Australia. Adult patients admitted to the General Medicine Unit during the six-month period from 5 February to 6 August 2024, with a principal diagnosis coded according to the *International Classification of Diseases, 10th revision, Australian Modification* (ICD-10-AM) U07.1 *(COVID-19, virus identified)* or U07.2 *(COVID-19, virus not identified)* were reviewed.

Patients were excluded if they were admitted directly to the ICU (as these patients fall outside routine General Medical workflows) or were critically unwell (where clinical decision-making is driven by sepsis management protocols), did not have a diagnosis of COVID-19 at the time of admission or were already receiving antibiotic therapy prior to presentation, including via general practitioner prescription or interhospital transfer (invalidating assessment of prescribing decisions at presentation), received antibiotics for a non-pulmonary indication (not relevant to suspected pulmonary bacterial coinfection), or were readmitted for the same COVID-19 infection (to avoid duplicate episodes).

A consensus recommendation for antibiotic therapy in patients admitted with COVID-19 was developed collaboratively by the Infectious Diseases and General Medicine teams. For the purposes of this recommendation, elevated total white cell count was defined as >$11.0\times \;10^{9}/L$ and elevated neutrophil count as >$7.5\times \;10^{9}/L$, reflecting standard institutional laboratory thresholds. The consensus recommendation was considered met if either an elevated total WCC with neutrophilia, or predefined radiological findings (lobar consolidation, lobar collapse, unilateral pleural effusion, or cavitation) were present. If no chest radiography had been performed at the time of admission, clinical findings were assessed for features suggestive of lobar consolidation or unilateral pleural effusion. The agreed consensus recommendation stated:

*“Reconsider antibiotic therapy unless there is high suspicion of concurrent bacterial infection, which may be indicated by either elevated total white cell count (WCC) with elevated neutrophil count, or radiological or clinical changes that cannot be explained by COVID 19 alone (e.g., lobar consolidation/collapse, unilateral pleural effusion, and/or cavitation)”.*

The audit comprised two periods. Cohort 1 (5 February–27 May 2024) represented the initial data collection phase of the audit cycle, and Cohort 2 (28 May–6 August 2024) represented the re-audit phase following targeted staff education. Educational interventions were delivered through:

(a) Presentations at Unit meetings, focusing on relevant instutitional guidelines and the consensus recommendation;
(b) Emails distributed to admitting clinicians detailing the consensus recommendation;
(c) Posters located in key clinical areas displaying the consensus recommendation; and
(d) Brief electronic reminder messages circulated to clinicians.

The educational intervention was informed by contemporary epidemiological evidence demonstrating a low prevalence of CABC in COVID-19 and by locally developed consensus in an area where published guidance is limited. Content was delivered consistently across multiple formats and focused on routine clinical scenarios encountered by admitting clinicians.

Patient records were reviewed using the hospital’s electronic medical record system. Extracted data included admission date, demographics, WCC and neutrophil count, relevant radiological findings, antibiotic administration, and predefined risk factors. Risk factors were classified across 16 predefined categories grouped into:

- Immunological and metabolic conditions—immunosuppression, medication-controlled diabetes mellitus, body mass index > 30 kg/m^2^, and hypertension.
- Organ dysfunction—estimated glomerular filtration rate < 60 mL/min/1.73 m^2^, chronic liver disease, respiratory compromise (emphysema, bronchiectasis, moderate to severe asthma, musculoskeletal or neurological disease), neurological conditions (stroke, dementia, demyelination), coronary artery disease, and heart failure or cardiomyopathy.
- Demographic or social factors—age > 50 years, residing in residential aged care, disability with multiple comorbidities and/or frailty, residence in a geographically remote area with reduced access to higher-level healthcare, pregnancy, and Indigenous background.

Two authors independently reviewed each episode, blinded to each other’s assessment. Results were subsequently compared, and discrepancies were resolved by consensus. Between-cohort differences were assessed using Fisher’s exact test (two-tailed, probability ≤ observed method) for small sample comparisons and the chi-squared ($\chi^{2}$) test when all expected cell counts were ≥ 5. Continuous variables were compared using the Mann-Whitney U test for non-normally distributed data and the two-sample t-test for normally distributed data.

The hospital network’s Human Research Ethics Committee determined that this audit was a Quality Improvement project and did not require Ethics and Governance approval.

## 3. Results

A total of 152 patient episodes were identified; 26 were excluded (readmissions, n = 4; already receiving antibiotic therapy, n = 8; ICU/critically unwell, n = 5; antibiotics for a non-pulmonary indication, n = 5; COVID-19 diagnosis not known at time of admission, n = 4), leaving 126 episodes for analysis. Cohort 1 included 80 episodes, and Cohort 2 included 46 episodes.

Baseline characteristics did not differ significantly between cohorts and there were no statistically significant differences between cohorts in premorbid functional status or illness severity (Table 1). As clinical reasoning could not be directly determined, adherence to the consensus recommendation was inferred from concordance with the specified criteria (WCC/neutrophil count and relevant radiological findings). Across both cohorts, 42 patients (33%) met these criteria for antibiotic therapy, while 31 (25%) received antibiotics; of these, 21 (68%) met the specified criteria.

**Figure 1:**
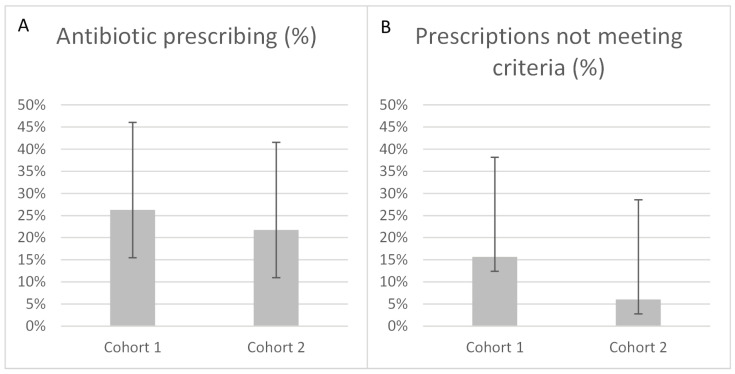
Antibiotic prescribing before (Cohort 1) and after (Cohort 2) targeted education. (**A**) Proportion of patients prescribed antibiotics. (**B**) Proportion of prescriptions not meeting consensus criteria. Error bars represent 95% confidence intervals.

In Cohort 1 (n = 80), 21 patients (26%) received antibiotics, of whom 13 (62%) met the specified criteria. In Cohort 2 (n = 46), 10 patients (22%) received antibiotics, of whom 8 (80%) met the specified criteria. There was no statistically significant difference between the two cohorts (Fisher’s exact test: *p* = 0.43). Among patients who did not meet the specified criteria, antibiotic prescribing decreased from 16% in Cohort 1 (n = 8) to 6% in Cohort 2 (n = 2), which also did not reach statistical significance (*p* = 0.30) (Table 2 and Figure 1).

Across both cohorts, 21 of 42 patients (50%) who met the specified criteria did not receive antibiotic therapy, and four of these went on to receive antibiotic therapy for respiratory infection within 48 h of admission. Two patients who did not meet the specified criteria and were not prescribed antibiotics on admission were prescribed antibiotics for a respiratory illness within 48 h (one in each cohort). None of the patients who did not receive antibiotics on admission but went on to receive antibiotics for a respiratory illness within 48 h required escalation in care to ICU or had a deterioration in their functional status at time of discharge.

## 4. Discussion

This closed-loop audit evaluated antibiotic prescribing for patients admitted with COVID-19 to the General Medicine Unit at a metropolitan hospital in South Australia. Following targeted education, antibiotic prescribing decreased from 26% to 22% of admissions. Among patients who did not meet the specified criteria, prescribing fell from 16% to 6%, while the proportion of prescriptions meeting the consensus recommendation increased from 62% to 80%. Although these differences did not reach statistical significance, confidence intervals were wide due to small cohort sizes and encompassed clinically meaningful improvements in antibiotic prescribing.

The observed rate of antibiotic use was substantially lower than many published reports [2,5,6,9,12], suggesting that stewardship principles are already being adopted within the unit. In the Australian context, there are few published studies for comparison, although a major hospital in Melbourne reported an antibiotic prescribing rate of 63.2% during a six-month period in 2020 [15]. Nevertheless, antibiotic use remained higher than the expected rate of CABC reported in numerous studies [5,6,7,8,9,10,11]. The smaller number in Cohort 2 reflected both the shorter data-collection period (71 vs. 113 days) and a lower rate of COVID-19 admissions (mean 4.5 vs 5.0 per week). The data-collection period was shorter due to logistical delays in implementing the targeted education and could not be readily extended due to a substantial staff change at the end of the six-month period.

The consensus recommendation was intended to support, not replace, clinician judgement. Additionally, it was designed to discourage antibiotic therapy when the specified criteria were not met, rather than to mandate prescribing when they were. This aligns with the finding that a substantial proportion of patients meeting the criteria were not prescribed antibiotics. The latest recommendations, even for patients with severe COVID-19, are to avoid antibiotics where the risk of bacterial infection is low [16]. However, at present there are no diagnostic tools that can consistently identify CABC. A range of biomarkers have been assessed — including C-reactive protein, WCC and differential, neutrophil:lymphocyte ratio, ferritin, and procalcitonin — but none fully substitute for clinical acumen [16,17,18]. When combined with radiological and clinical findings, however, they can assist clinicians in reducing unnecessary antibiotic use [16,18,19,20,21].

On exploratory post hoc review, several potential refinements to the current consensus recommendation were identified. First, the requirement for a neutrophilia was redundant, as all patients with an elevated total WCC also had a corresponding neutrophilia. Second, approximately half of patients meeting radiological criteria had only borderline or minor findings, such as a small unilateral pleural effusion, which may not indicate bacterial infection. Third, the clinical context remains critical: for example, those with incidental or mild illness and no definitive evidence of bacterial infection are unlikely to be harmed by initial omission of antibiotics, even if it later becomes apparent that a bacterial coinfection is present, provided there is adequate patient monitoring and opportunity for clinical re-review.

These exploratory observations suggest that a more stringent definition may further reduce antibiotic use. In this cohort, 24 out of 126 patients (19.0%) would have met these revised criteria, compared with 42 patients (33.3%) using the original definition. A potential future recommendation could therefore state: “*Reconsider antibiotic therapy unless there is at least moderate illness severity and high suspicion of bacterial coinfection, which may be indicated by an elevated WCC or significant radiological or clinical changes that cannot be explained by COVID-19 alone (e.g., lobar consolidation/collapse, moderate or larger unilateral pleural effusion, or cavitation)*”. This approach may provide more stringent guidance while maintaining clinician autonomy.

Strengths of this audit include its closed-loop design, multidisciplinary consensus, and focus on practical, locally implementable stewardship strategies. Limitations include the small cohort size, single-site design, lack of direct measurement of educational impact with reliance on retrospective inference of prescribing rationale, and reliance on published rates of CABC instead of evaluating the rate at our institution directly. The retrospective design introduces potential for information bias and residual confounding, although predefined criteria and independent review were used to mitigate these limitations.

Diagnostic uncertainty at the time of admission also remains a challenge, as CABC is usually confirmed only retrospectively from sputum or blood culture results, which take time to finalise and may be negative if antibiotics have already been commenced. Routine microbiological assessment for CABC is further constrained by the fact that the full range of diagnostic tests for bacterial pneumonia is not routinely applied to patients presenting with COVID-19. Moreover, in many pre-COVID-19 studies investigating the aetiology of pneumonia, more than half of patients with clinical and radiological features of infection had no causative organism identified [22]. Consequently, studies attempting to estimate the prevalence of CABC based on microbiological diagnosis are likely to underestimate the true rate of bacterial co-infection.

## 5. Conclusions

Although statistical significance was not reached, the findings suggest that targeted education and clear, context-specific guidance have potential to improve antimicrobial stewardship. Ongoing audit cycles, continued education, and iterative refinement of the consensus recommendation may further reduce unnecessary antibiotic use.

## Figures and Tables

**Table 1:** Baseline characteristics and antibiotic use by cohort.

Characteristic	Cohort 1	Cohort 2	*p* Value
Total number	80	46	–
Median age (IQR), years	81 (72.8–86.0)	82 (70.3–86.0)	*p* = 0.84 ^1^
Males (% total)	36 (45%)	28 (61%)	*p* = 0.09 ^2^
Mean number of risk factors	4.3	4.3	*p* = 0.86 ^3^
**Premorbid functional status**			*p* = 0.52 ^2^
Independent (%)	25 (31%)	17 (37%)	–
Supported at home (%)	48 (60%)	23 (50%)	–
Residential care (%)	7 (9%)	6 (13%)	–
**Illness severity**			*p* = 0.45 ^2^
Incidental or mild (%)	35 (44%)	24 (52%)	–
Moderate (%)	37 (46%)	16 (35%)	–
Severe or critical (%)	8 (10%)	6 (13%)	–

IQR = interquartile range; *p* values for baseline characteristics are provided for descriptive comparison only. ^1^ Mann–Whitney U test; ^2^ χ^2^ test; ^3^ Two-sample *t*-test.

**Table 2:** Antibiotic prescribing for CABC and adherence to consensus criteria, by cohort.

	Cohort 1	Cohort 2	*p* Value	Absolute Difference (95% CI)
**Patients prescribed antibiotics** ^a^, n	**21** **26.3%**	**10** **21.7%**	**0.57** ^1^	**4.5%** (95% CI: –10.8% to 19.8%)
– Met specified criteria ^b^, n	13 61.9%	8 80.0%	0.43 ^2^	–
Patients not meeting criteria ^a^, n	51 63.8%	33 71.7%	–	–
– **Prescribed antibiotics** ^c^, n	**8** **15.7%**	**2** **6.1%**	**0.30** ^2^	**9.6%** (95% CI: –3.3% to 22.5%)

CABC = community-acquired bacterial coinfection; CI = confidence interval. ^1^ χ^2^ test. ^2^ Fisher’s exact test. ^a^ Of entire cohort. ^b^ Of patients prescribed antibiotics. ^c^ Of patients not meeting the specified criteria.

## Data Availability

The data that support this study cannot be publicly shared due to ethical reasons.

## Abbreviations

The following abbreviations are used in this manuscript:

AMR	Antimicrobial resistance
CABC	Community-acquired bacterial coinfection
COVID-19	Coronavirus disease 2019
ICU	Intensive care unit
ICD-10-AM	International Classification of Diseases, 10th revision, Australian Modification
IQR	Interquartile range
WCC	White cell count
